# Experience of Families During Admission of Their Minors to a Paediatric Intensive Care Unit: A Phenomenological Study

**DOI:** 10.1111/scs.70055

**Published:** 2025-06-23

**Authors:** Patricia Rubio‐Garrido, Leticia Bazo‐Hernández, Anna Enrich‐Font, María Francisca Jiménez‐Herrera

**Affiliations:** ^1^ Department of Nursing Rovira i Virgili University Tarragona Spain; ^2^ Department of Nursing Vall d'Hebron University Hospital Barcelona Spain; ^3^ Department of Nursing Borås University Borås Sweden

**Keywords:** child, family‐centred care, hospitalised, intensive care units paediatric, perception, phenomenological study

## Abstract

**Background:**

Being admitted to a paediatric intensive care unit is a stressful situation for the minor and their family, causing emotional alterations that generate changes in psychological, physical and social aspects, as well as on how to take care of the minor.

**Aim:**

The objective of this study was to explore the coping strategies experienced by families who have a minor admitted to the paediatric intensive care unit.

**Methods:**

Phenomenological qualitative research study. A sample of 12 participants was obtained from families who had a prolonged admission to the paediatric intensive care unit of a minimum duration of 3 months and who were not in an end‐of‐life situation. The data analysis was carried out by deepening the study theme during the data collection of the three group interviews conducted.

**Ethics Statement:**

This study is part of a predoctoral research project approved by the Ethics Committee of the Vall d'Hebrón University Hospital in Barcelona (approval code: PRAMI‐273/2015).

**Results:**

Two main categories were identified that emerged from the families' perceptions related admission of their minors to the paediatric intensive care unit and their subsequent adaptation to the new situation: (1) Family self‐perception: the families' own perceptions of different feelings and emotions experienced during admission and their interpretations are collected. (2) Role of the caregiver: understood as the families' perceptions of the capacity and willingness to face the new situation in the child's health‐disease process; and 10 subcategories: training, comfort, confidence, fear, anxiety, impotence, loneliness, will, adaptability and reference family group.

**Conclusions:**

The findings show the need to carry out an integrative approach to families which coexist in a paediatric intensive care unit; through the training of these families to be able to cope with health changes.

## 1 Introduction

Children from newborn to 18 years of age are admitted to a Paediatric Intensive Care Unit (PICU) due to infections, respiratory and cardiac diseases, trauma, among other conditions. Admission to the PICU represents a highly stressful experience for the family, affecting their well‐being due to uncertainty about the prognosis, separation and difficulties in emotional bonding. In addition, financial and logistical barriers exacerbate the situation, causing caregivers anxiety, depression, stress, which impacts on their psychological, physical and social well‐being, as well as the way they care for the child. Support and effective communication with healthcare professionals is essential to address this situation and ensure the best care for the critically ill child [1, 2, 3, 4, 5, 6].

In recent decades, technological advances in healthcare have contributed significantly to the increase in life expectancy. As a consequence, there has been an increase in the number of patients with multiple pathologies or complex clinical conditions, which can prolong hospitalisation time and require specialised care. In this context, it is essential to actively incorporate the main caregiver in the care process of the critically ill child, promoting his or her adaptation to the hospital environment and favouring more humanised care [3, 5].

Currently, PICUs focus on the Family‐Centred Care (FCC) model, where family members are actively involved in the care of the child. This approach promotes adaptation to the patient's health status and the development of strategies to address family needs [6]. It is important to consider the caregiver as a key figure in the child's treatment, as their emotions and experiences can influence their recovery [3, 7].

One of the main consequences of prolonged admission to the PICU is the development of PICS‐F, which causes adverse psychological reactions such as anxiety, acute stress disorder, depression, pain, sleep problems and poor nutrition. These conditions affect the ability of families to fulfil their role as caregivers [1, 2, 6, 7, 8, 9, 10, 11, 12]. Therefore, it is essential to investigate their needs and emotions in order to improve nursing care and facilitate their adaptation to the PICU.

A study by Oxley, which analyses the experience of parents with children admitted to PICU, concludes that the emotions of family members vary throughout the process and may extend even after hospital discharge [4]. In this sense, social support, effective communication with the healthcare team, and, in particular, with the nurses, can mitigate the negative psychological impact on families. Likewise, the use of clear and accessible language, avoiding medical terminology, contributes to reducing stress levels [1, 4, 7, 8].

In the last decade, several coping strategies for parents have been identified. Curtis et al. suggest the implementation of the VALUE communication model, which improves interaction between medical staff and families of PICU patients [13]. Another effective strategy is the use of a reflective diary, which allows caregivers to record their experience, concerns, and emotions during hospitalisation. This tool helps to reduce anxiety and stress, as well as serving as a means of communication between the family and the healthcare team [2, 5, 10, 11, 14, 15].

Finally, the creation of support groups among families has proven to be a valuable resource in the recovery of caregivers. Interaction with other families who have gone through similar situations fosters empathy, exchange of advice and mutual support, strengthening the emotional well‐being of caregivers [11, 12, 13, 14, 15, 16].

## 2 Purpose

The objective of this study was to explore the coping strategies experienced by families who have a minor admitted to the paediatric intensive care unit.

## 3 Methodology

### 3.1 Design

The research design of this study is based on qualitative methodology. For this purpose, a descriptive phenomenological study is carried out, which delves into the experiences and perceptions of families in a paediatric intensive care unit.

### 3.2 Setting and Sample

This study was conducted by PICU nurses to examine the experiences of families with children who had been admitted to the unit. The data collection period was from January to March 2020 in the PICU of the Vall d'Hebrón University Hospital in Barcelona.

Participants in this study were family members of children who had some PICU experience. Participants were contacted for identification and collectionvia telephone, control visit, or hospital admission, and who wished to participate in family group sessions in which they could share their experiences.

A total of 12 participants who had a prolonged PICU admission of at least 3 months, and who were not in an end‐of‐life situation were gathered.

### 3.3 Data Collection

The study environment was developed in the Vall d'Hebrón's University Hospital “Ciberaula” (online platform), used as a space for group sessions. Prior to the group sessions, informed participants were asked to consent, and they were offered the possibility of participating in any of the different sessions that were to be held. Participating families were informed of the entire development of the study at all times. Participants voluntarily accepted to participate and had the option to withdraw at any time from the study. To avoid possible biases derived from the emotional bonding that is generated after a prolonged admission to the unit between the researchers and the participants, the research team confirmed that no emotional bonding had been established with the participants. The group interviews were conducted by the researchers, who remained impartial during the sessions and with a moderator who allowed the participants to express their experiences and perceptions of their admission to the PICU. The interviews were followed using a semi‐structured script with open‐ended questions based on the literature to deepen the topic of study, and were modified to integrate new ideas in order to generate a reflective process of humanization of care in the PICU.

The main research question answered: How do the patients' families live in the PICU? giving the possibility of being able to express their opinion to the participants in an orderly manner and being able to discuss the perceptions in the PICU as a group. The group interviews had an average duration of 53 min. The group sessions were held until data saturation was achieved. All interviews were recorded, transcribed and were accessible to participants.

### 3.4 Data Analysis

To conduct this descriptive study with a phenomenological analysis of the lived experiences of families admitted to the PICU, we followed a rigorous analytical process to gain an in‐depth understanding of these experiences. First, we transcribed the interviews verbatim and conducted repeated readings of the narratives to capture their overall meaning. We then identified units of meaning within the transcripts, which we coded and grouped into emerging thematic categories. From these categories, we developed central themes that encapsulated the essence of the lived experience. These themes were then interpreted and synthesised into a coherent narrative, framed within phenomenological theory and supported by existing literature.

To ensure the reliability of the study, we employed several strategies to strengthen the rigour of our analysis. Researcher triangulation allowed us to analyse the data independently and compare our interpretations. In addition, we applied data saturation as a criterion for determining when to stop data collection, ceasing once no new meanings or categories emerged. We also incorporated an external audit, in which an expert reviewed the consistency of our coding process. Verbatim quotes from participants were included to support our findings and ensure authenticity. In addition, we kept a research diary to document our reflections and possible biases, ensuring that the analysis remained as objective as possible. Through these strategies, we provided an accurate and rigorous interpretation of families' experiences in the PICU.

Data analysis was conducted using written transcripts of the group sessions, following Colaizzi's model [17]. To ensure a rigorous and systematic analytical process, an in‐depth transcription of the interviews was conducted immediately after each session to maximise data collection and allow for detailed analysis. We then conducted a thorough reading of the transcripts, employing an inductive approach to identify the most relevant themes related to the phenomenon under study. Common categories and subcategories were identified among the participants, and a description of the phenomenon was developed based on these themes and the participants' perceptions. Finally, the data were verified and coded by the lead researcher. For reliability purposes, another researcher independently categorised the themes derived from the interviews and compared them with the original coding to improve consistency and validity. Throughout the analysis, we ensured that there was concordance between the participants' excerpts, their perspectives and our research team's interpretations. This process strengthened the consistency of the findings. Codes, categories and subcategories were thoroughly reviewed in relation to all data collected in the sessions.

To guarantee the rigour of this phenomenological study, the quality criteria proposed by Calderón [18] in qualitative health research were applied. First, the epistemological adequacy of the families' experiences was ensured by using qualitative techniques such as in‐depth interviews. Secondly, the importance and relevance of the data was considered, given that the testimonies collected provide valuable information for the improvement of family‐centered care and the humanization of PICU care. Third, reflexivity was incorporated throughout the process through constant self‐reflection by the research team and triangulation of perspectives, thus minimising possible biases in the interpretation of the data. Finally, the validity of the study is guaranteed through rigorous analysis based on the families' accounts, as well as through internal coherence between the results obtained and the experiences expressed, allowing a deep and useful understanding for clinical practice.

This study is part of a predoctoral research project approved by the Ethics Committee of the Vall d'Hebrón University Hospital in Barcelona (approval code: PRAMI‐273/2015). Permission was granted by the care management of the Vall d'Hebrón Hospital. Firstly, all health professionals of the Paediatric Intensive Care Unit were informed. Secondly, informed consent was obtained from all participants prior to the group sessions. Finally, the study participants were informed at all times of the research process, the confidentiality of the data and the willingness to participate in the study, and the possibility to withdraw at any time.

## 4 Findings

All family participants identified themselves as female, aged between 19 and 50 years, with the most predominant age range being 30–35. The most prevalent marital status was married, followed by separated and single. Their current employment status was mainly low employment. Finally, the predominant level of education was university studies. The children in the participating families were aged between 1 month and 14 years, with a reserved diagnosis without being terminal and a minimum experience of 3 months of admission to the PICU.

From the analysis of the interviews based on those proposed in phenomenological research [19], two categories and a total of 10 subcategories were identified, as shown in Table 1.

**TABLE 1:** Categories and subcategories.

Categories	Subcategories	Number of sentences
1. Family self‐perceptions	1.1. Training	9
	1.2. Comfort	10
	1.3. Trust	3
	1.4. Fear	13
	1.5. Anxiety	6
	1.6. Impotence	6
	1.7. Loneliness	4
2. Role of the caregiver	2.1. Will	7
	2.2. Adaptability	11
	2.3. Referral family group	5

These categories arose from the perceptions of the families during admission to the PICU of the Vall d'Hebrón Hospital, and their subsequent adaptation to the new situation. The two main categories are family self‐perceptions and the role of the caregiver.

### 4.1 Category 1: Family Self‐Perceptions

This category encompasses the families' perceptions of the different feelings and emotions they experienced during admission to the PICU, as well as their personal interpretations of these. Within these emotional experiences, key aspects were identified as the training process, where family members acquired knowledge about the patient's condition and care; the feeling of comfort, derived from the support received from the healthcare staff; and trust, both in the healthcare professionals and in their own ability to cope with the situation.

On the other hand, negative emotions also emerged, such as fear of the uncertainty of the child's state of health, anxiety about the evolution of the treatment, helplessness in feeling limited in their actions, and loneliness, especially at times when they could not be physically present with their loved one. These emotions reflect the complexity of the families' experience and its impact on their emotional well‐being.

#### 4.1.1 Training

Various emotions and feelings associated with the process of empowerment and capacity building of families after admission to the PICU were identified. This empowerment refers to how, from a difficult and stressful experience, family members developed new skills, gained knowledge about their child's health condition, and felt more prepared to cope with the situation. Some of the expressions collected in the testimonies reflect how, despite the initial fear and uncertainty, the relatives gained confidence in themselves, in the medical team, and in their own role in the care process.

PS4: … that they have to have the strength to fight. One day you're down, and you cry, and then you're back again, fighting. That's what you must do.

In general, expressions of struggle are observed, of moving forward, of greater personal preparation, of pride, among others. Another example:

PS5: … I felt very bad for my son. But it could be worse, couldn't it? I don't think so. I'm ready to see him as he was during the first intervention.

Shows greater strength to move forward.

#### 4.1.2 Comfort

We understand this subcategory as the feelings of tranquillity and security that the participants felt both related to the unit and to their child. Expressions such as:

PS2: if it weren't for the way they look at me, I wouldn't… I would have broken down a long time ago.

PS1: ICU …. it's were i'm comforted, where I'm calm, that… where I know that nothing is going to happen to her.

Receiving gratitude from the child helps families to not give up in the face of adverse situations. When gratitude is expressed, a strong human connection is created that fosters a positive, two‐way atmosphere.

#### 4.1.3 Trust

That feeling of hope, of security towards other people in this case with the staff of the PICU. Comments such as:

PS10: … For me, the ICU is my second family… but it is true that, there are people to whom their profession reaches more than others, but that is why I keep saying that the ICU has a great team of people and nothing else.

PS5: Positive, I want to say of the staff, of the treatment towards me, towards my son, it has been perfect.

The bond of security that is generated towards the professionals increases with the time of admission and generates positive feelings of adaptation. The expressions reported by the mothers show the high value that is given to the multidisciplinary group.

#### 4.1.4 Fear

This subcategory includes that emotion generated by a situation of danger, real or hypothetical, present or future, which can block, generate respect, and distrust towards an event. It is the subcategory where we can find the largest number of comments. Some of the expressions included are:

PS9: Yes, yes. I'm afraid of losing her, but I also have faith.

PS10: The therapist tells me that fears are to be faced… I did get scared, that's why I asked for psychological help. Yes, for me, therapy is helping a lot. I was very afraid of losing my son and that fear still remains with me…

PS3: Well, going out on the street was more scary, afraid of infections and things like that

Everyday situations such as going out or sleeping are perceived as risky activities due to the child's fragility, without taking into account the child's development. Social interactions are essential for the child's well‐being and hinder emotional development and social skills.

#### 4.1.5 Anxiety

This feeling maintains a relationship with the previous one, since it is understood as that feeling of fear or restlessness related to a situation of uncertainty, a danger that makes you restless, tense. Family members express it as:

PS3: I had physical symptoms, I had a lot of anxiety.

PS6: … you stay in this state of… and you really don't know who your enemy is, do you? That's it, it's very hard, this is very hard.

The situation of vulnerability that they perceive due to the child's condition causes anxiety that can lead to situations perceived as threatening or stressful. Anxiety must be addressed through the natural responses that the human body has to situations perceived as negative.

#### 4.1.6 Impotence

This subcategory is defined as the feeling of lack of power, ability to be able to do things, in this case, feelings of guilt, comparing children to others, wanting perfect lives… feelings of not being able to do everything. It is reflected in:

PS6: … They don't understand it, not because everything happens to me, because this is intense.

PS7: … We do not want the feeling of suffering ourselves. We want perfect and happy lives and many times these things happen, and you have to consider life in a different way.

PS5: … The only fact that reminds you day after day of living at home with this disease is the medication and the fact that your child is not up to speed with others. Sorry. (Crying)

Emotional experiences are intense and variable, just like the stages of health, and can sometimes lead to a situation of sudden change. The inability to handle situations has a negative effect on coping and does not allow positive aspects to be seen. The underlying causes must be identified in order to work towards achieving objectives. It is important to approach communication in a clear and open way and from empathy to avoid problems of understanding.

#### 4.1.7 Loneliness

The admission of a minor to the PICU can generate feelings of loneliness towards family members related to the fact of being alone since they come from other communities and do not have close family support, or also because you feel alone with the minor's illness. Expressions like the following show it:

PS2: And I'm the one who has to give encouragement and it should be the other way around, but no… I don't know, in that aspect, for example, I feel alone…

PS6: … But… but also I have always lacked the support to say… I don't know, as the two mom's say, this is not understood from outside. My story is very different from the two of them, although I feel shattered, but shattered and… I need support, I think it will be very good for me.

Admission is seen as an emotional challenge. The need to feel in company is rooted in our social nature. Emotional interactions are essential for emotional well‐being.

### 4.2 Category 2: Role of the Caregiver

This category includes the perceptions of families about the capacity and willingness to face the new situation in the child's health‐disease process, and also new lines of action, where we find: will, adaptability, reference family group.

#### 4.2.1 Will

Here we find reflected the ability/feeling of being able to say that they are able to carry out special/different care for their child after a prolonged admission to the PICU. Phrases such as:

PS2: also when I was going to leave, everyone was asking me if I was sure, that … are you ready to go home, I felt very prepared…

PS6: No, none…you become an expert, loving him so much seeing him do so well, …

PS7: … The whole family has to be very close to be able to endure this process, because it is not easy, it is not easy.

Nursing professionals have the mission of instructing families and generating a predisposition to accept the new role of caregiver, adapting to the needs of the child. Training should be evaluated through clarification during admission so that it is experienced as a positive aspect and the complexity should be highlighted.

#### 4.2.2 Adaptability

This subcategory is mentioned a lot by families, addressing their ability to adapt to the new situation, either going back home, with mediation, care, or as a record of the process lived to explain both to the child in the future and to the world. Expressions such as the following reflect this.

PS2: at first, yes, I organized so I could be with my mother and my mother‐in‐law while her father worked, but… I lost the fear, and now I'm by myself.

PS10: I personally am a person who likes to participate a lot in the care of my son. And I like to enjoy the bath afterwards, they taught me to do the fixings of the probes on the nose.

PS12: I mean, it's totally different how I feel when I'm here or when I'm at home. I mean, when I'm at home there's normalcy.

PS3: One day here at ICU, I said: I'm going to write, I do have a diary, and it's my treasure. Yes, I do have a diary, but telling M

Respecting the times improves adaptation to the new state, through trust. The difficulty in asking for help is usually caused by suffering a high level of emotional stress due to a change and/or isolation from the environment, an unconscious blockage of acceptance.

The search for self‐help strategies is shown in families as a good coping resource. It is necessary to know how to seek and ask for help.

#### 4.2.3 Reference Family Group

In this subcategory we wanted to collect the thoughts related to a proposal for a line of action where there was an expert family member(s) to talk with other families who are going through situations similar to current families and provide support among them. This proposal was well received by families, as shown below:

PS9: Yes, we all look for it innately, I belong to a group of parents with heart disease. I met PS12. Thanks to that and other things, it helps me a lot. All that support of parents of children who have had these experiences give me that parents give me…

PS10: … But I offer my time here, to every person who is here and I want to share my friendship with them, because bring together is not the same as being it alone, you know.

PS8: I also remember the day DA entered ECMO, and I remember the two of them being involved with me with MJ, well, that's where the ideas of doing that family therapy meeting was born.

Health professionals are the fundamental pillar of training. They must instruct and train families to help them develop themselves, so that they can serve as inspiration. Families seek to be able to share their experiences, as a method of support and accompaniment.

## 5 Discussion

The objective of this study was to identify the emotions experienced by families who have a minor admitted to the PICU. The families that participated identified: family self‐perceptions and the figure of the caregiver. Our research highlighted the importance of the integration of the family within the child's health care, in order to train them and avoid maladaptation after admission to the PICU.

The phenomenological analysis allowed us to understand the family as an essential nucleus in the health field with respect to the care of the child, from a biopsychosocial perspective with emphasis on the phenomenon of study as part of the treatment and disease process.

The families expressed a series of stages of emotions and feelings, related to the child's health process. They identified through self‐perceptions, feelings such as empowerment, security, tranquillity, confidence, fear, anxiety, loneliness and impotence. These emotions fluctuate throughout the child's admission, making it crucial to provide targeted interventions that foster emotional resilience.

Lazarus R and Folkman S talk about how the human being is able to generate different coping strategies for lived experiences, whether understood by the individual as a positive or negative experience. This is how families express it during their stay in the PICU, allowing them to experience the adaptive process in order to achieve the emotional balance necessary for excellent care [20]. Coping strategies can be enhanced by healthcare providers through psychoeducation, emotional support groups, and mindfulness practices. Teaching families relaxation techniques or guiding them in cognitive reframing can help mitigate distress and promote well‐being.

The literature shows that, in most families, significant changes occur in the emotional state, generating a change in the behaviour pattern that sometimes makes it difficult to manage emotions and adapt to the new state of health [4, 21, 22, 23, 24, 25]. Therefore, healthcare teams can implement regular family check‐ins, providing a safe space for caregivers to express concerns and receive guidance. Regular debriefing sessions or structured family meetings can foster open communication and emotional processing.

It should be noted the importance that health professionals have in identifying and helping families adapt in the setting of a PICU [26, 27]. The results of this research reflect experiences that underscore the importance of preserving the mental health of the child's primary caregiver, with the goal of optimising coping strategies. Providing access to mental health resources, such as on‐site counsellors or referral services, can be invaluable in strengthening family resilience.

Regarding the figure of the caregiver, families expressed the need to participate in care, seeing this need as an emergent act by nature of the family's affective bond. Through the capacity for adaptation related to the willingness to benefit the child, strategies for improvement in care and the family's adaptive process are generated. Healthcare providers can encourage this involvement by offering hands‐on training in caregiving tasks, clear care protocols, and opportunities for families to participate in decision‐making processes.

Studies carried out on the integration of the family in the PICU confirm the needs demanded by the relatives regarding the specific care of the minor, such as support and social impact [6, 15, 28, 29]. Highlighting the integration of the family as a need for improvement during admission, to favour adaptation after discharge [30].

To implement FCC principles in practice, healthcare teams can adopt structured strategies like creating personalised care plans with the family, involving families in interdisciplinary rounds, and establishing family resource centers within the PICU. These actions not only empower caregivers but also reinforce their role as active participants in the child's healing process.

Ultimately, due to the nature of the changes experienced in the health status of the minor, there is a need to accompany, instruct, and enhance the figure of the family member by health personnel. Through the incorporation of the family in health care during health admission, it provides the opportunity to improve the adaptive process of the child's new state of health.

In conclusion, by fostering emotional support, equipping families with coping tools, and integrating them fully into the care process, healthcare providers can create a nurturing PICU environment that not only enhances the child's recovery but also promotes long‐term family well‐being. This holistic approach bridges the gap between clinical care and emotional support, recognising the family as a vital partner in the healing journey.

### 5.1 Strengths and Limitations

For this study, participants with experience of families having prolonged admission of their minor in PICU and who were not in an end‐of‐life process were selected. In future research, it could be extended to other critical units and include health professionals in order to deepen health humanisation. It is important to note that the participants had been in a humanised intensive care unit where family participation is essential for the approach to patient care, this factor may be limited by the subjective perception of care.

### 5.2 Clinical Implications

The findings of this study have important clinical implications in the context of the PICU, as they demonstrate the need to integrate the family as an active agent in the care process through the implementation of FCCs. The experiences collected in the two categories: family perceptions and caregiver role, have a direct impact on the quality of care.

From a clinical perspective, understanding the range of emotions experienced by families, such as fear, anxiety, helplessness, but also confidence and comfort, allows professionals to adapt their emotional support and design interventions aimed at mitigating parental stress. Continuous training of families improves their confidence and participation, which favours adherence to treatment and the development of coping strategies during hospitalisation.

In addition, recognition of the active role of the caregiver highlights the need to encourage their involvement from admission, promoting institutional programmes that strengthen family resilience and facilitate the transition to discharge. This evidence reinforces the importance of PICUs evolving towards more humanised models, where family‐health care team interaction is integrated as a key indicator of quality of care.

## 6 Conclusions

The aim of this study was to explore the coping strategies used by families with a minor admitted to the PICU. From the results obtained, we can conclude that families go through a complex emotional process, characterised by feelings of fear, anxiety, helplessness and loneliness. These feelings highlight the urgent need for intervention by healthcare professionals, who must be trained to identify the families' coping strategies, anticipate their needs, and facilitate their adaptation to the process of health and illness.

Several coping strategies were identified among families. Firstly, many of them develop empowerment mechanisms, which enable them to strengthen their capacities to cope with their child's hospitalisation. The training provided by health staff plays a key role, as it allows families to better understand the situation and to actively participate in the care of their child. Likewise, trust in health professionals and in the treatments applied generates a sense of greater security and control in the midst of uncertainty.

Another relevant finding was the importance of emotional and social support in the coping process. Families resort to the support of other family members, the accompaniment of the health team, or external support networks to cope with the experience. However, feelings of loneliness and helplessness were also evident, especially when families perceive limitations in their capacity for action or access to the child.

The study also highlights that families facing the admission of a child to the PICU are particularly susceptible to difficulties in adapting to the change in their child's health status. This process highlights the need for health professionals to intervene early and effectively to support families in their adjustment process.

Furthermore, it concludes that it is essential to adopt an integrative approach in the approach to families, including both the training of health professionals and the families themselves. This training should focus on providing them with tools to deal with changes in the child's health and alterations in their daily routines. The study also highlights the importance of further research on the role of families within the healthcare team, as their role is becoming increasingly crucial within the healthcare system.

On the other hand, the growing socio‐cultural diversity in health care means that the demands of families are increasingly more specific, complex and, at the same time, individualised. This process of adaptation improves families' ability to cope with changes in their child's state of health, favouring a more effective and personalised response to each situation.

In conclusion, the coping strategies of families in the PICU vary according to their personal resources, the support available, and their degree of participation in the hospitalisation process. These findings underline the need to provide comprehensive support to families, strengthening both their emotional well‐being and coping skills in order to improve their experience during their child's admission to the unit.

## Author Contributions

The authors Patricia Rubio‐Garrido and Anna Enrich‐Font collaborated in the collection of data and drafting of the manuscript; and Leticia Bazo‐Hernández and María Francisca Jiménez‐Herrera participated in the preparation of the manuscript. All have participated in the review and approval of the article, respecting the ethical‐legal standards in force, and approving the final version of the article.

## Disclosure

This article contributes to the dissemination of the main author's doctoral thesis.

## Ethics Statement

This study is part of a predoctoral research project approved by the Ethics Committee of the Vall d'Hebrón University Hospital in Barcelona (approval code: PRAMI‐273/2015).

## Conflicts of Interest

The authors declare no conflicts of interest.

## Data Availability

The data that support the findings of this study are available from the corresponding author upon reasonable request.
